# Supplementary material for: Identification of photoperiod-induced specific miRNAs in the adrenal glands of Sunite sheep (Ovis aries)
Source: Front Vet Sci. 2022 Jul 22;9:888207. doi: 10.3389/fvets.2022.888207 (PMC9354845; doi:10.3389/fvets.2022.888207)
Supplement: Supplementary file 1 [file Data_Sheet_1.ZIP › Supplementary file/miRDeep2 file.zip!/miRDeep2 file/Know_Structure/SP42A2/oar-mir-29b-1.pdf]

miRBase precursor : oar-mir-29b-1
Total read count : 14311
oar-miR-29b read count : 13332
remaining reads : 979

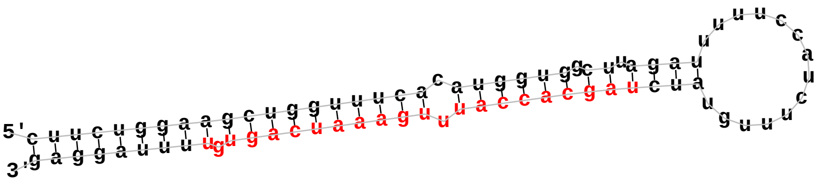

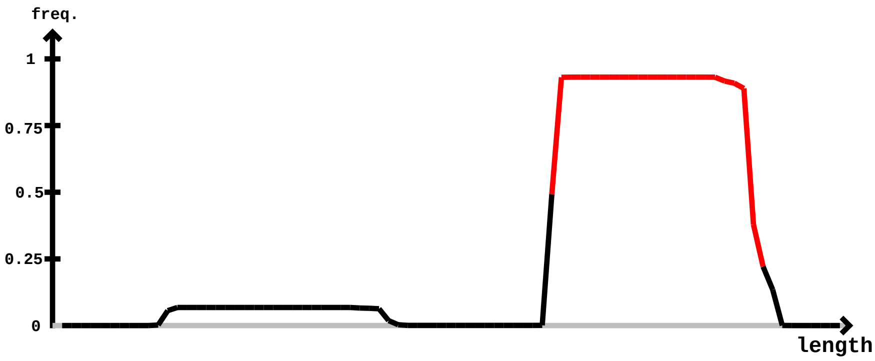

oar-miR-29b

```
5'- cuucuggaagcugguuucacaugguggcuuagauuuuuccaucuuuguaucuagcaccauuugaaaucaguguuuuaggag   -3'    exp
    (((((((((((((((((((.((((((.((.((((..............)))))))))))).)))))))))).)))))))))    reads      mm         sample
    .........gcugguuucaUauggugg......................................................    1          1          seq
    .........gcugguuucacaugguggcuu...................................................    7          0          seq
    .........gcugguuucacaugguggcuua..................................................    5          0          seq
    .........gcugguuucacaugguggcuuag.................................................    1          0          seq
    .........gcugguuucacaugguggcuuaga................................................    16         0          seq
    ..........cugguuucacaugguggcuu...................................................    20         0          seq
    ..........cugguuucacaugguggcuua..................................................    10         0          seq
    ..........cugguuucacaugguggcuuag.................................................    10         0          seq
    ..........cugguuucacaugguggcuuaga................................................    549        0          seq
    ..........cugguuucacaugguggcuuagau...............................................    162        0          seq
    ..........cugguuucacaugguggcuuagauu..............................................    20         0          seq
    ...........ugguuucacaugguggcuu...................................................    1          0          seq
    ...........ugguuucacaugguggcuuag.................................................    15         0          seq
    ...........ugguuucacaugguggcuuaga................................................    77         0          seq
    ...........ugguuucacaugguggcuuagau...............................................    59         0          seq
    ...........ugguuucacaugguggcuuagauu..............................................    17         0          seq
    ............gguuucacaugguggcuuaga................................................    1          0          seq
    .................................uuuuuccaucuuuguauc..............................    7          0          seq
    .................................uuuuuccaucuuuguauU..............................    1          1          seq
    .................................................ucuagcaccauuugaaau..............    1          0          seq
    .................................................ucuagcaccauuugaaauc.............    1          0          seq
    .................................................ucuagcaccauuugaaaucagu..........    3          0          seq
    ..................................................cuagcaccauuugaaau..............    1          0          seq
    ..................................................cuagcaccauuugaaaC..............    1          1          seq
    ..................................................cuagAaccauuugaaau..............    1          1          seq
    ..................................................cuagcaccauuugaaauc.............    88         0          seq
    ..................................................cuaCcaccauuugaaauc.............    1          1          seq
    ..................................................cuagcaccauuugaaaucG............    1          1          seq
    ..................................................cuagcaccauuugaaauca............    31         0          seq
    ..................................................cuagcaccauuugaaaucGg...........    2          1          seq
    ..................................................cuagcaccauuugaaaucag...........    62         0          seq
    ..................................................cuagcaccauuugaaaucagu..........    4197       0          seq
    ..................................................cuagcaccauuugaaaucGgu..........    33         1          seq
    ..................................................cuagcaccauuugaaaucagug.........    1705       0          seq
    ..................................................cuagcaccauuugaaaucagugu........    909        0          seq
```

oar-miR-29b

```
cuucuggaagcugguuucacaugguggcuuagauuuuuccaucuuuguaucuagcaccauuugaaaucaguguuuuaggag
..................................................cuagcaccauuugaaaucaguguu.......   4      0    seq
...................................................uagcaccauuugUaauc.............   1      1    seq
...................................................uagcaccauuugaaauc.............   104    0    seq
...................................................uagcaccauuugaaauU.............   11     1    seq
...................................................uaUcaccauuugaaauc.............   1      1    seq
...................................................uagcaccauuugaaauA.............   1      1    seq
...................................................uagcaccauuugaaaucG............   45     1    seq
...................................................uagcaccauuugaaauca............   35     0    seq
...................................................uagcaccauuugaaaucag...........   62     0    seq
...................................................uagcaccauuugaaaucGg...........   154    1    seq
...................................................uagcaccauuugaaaucGgu..........   868    1    seq
...................................................uagcaccauuugaaaucagu..........   2207   0    seq
...................................................uagcaccauuugaaaucagug.........   547    0    seq
...................................................uagcaccauuugaaaucagugu........   329    0    seq
...................................................uagcaccauuugaaaucaguguu.......   1910   0    seq
...................................................uagcaccauuugaaaucaguguuu......   5      0    seq
...................................................uagcaccauuugaaaucaguguuuu.....   1      0    seq
....................................................agcaccauuugaaaucag...........   1      0    seq
....................................................agcaccauuugaaaucagu..........   1      0    seq
....................................................agcaccauuugaaaucGgu..........   1      1    seq
....................................................agcaccauuugaaaucaguguu.......   6      0    seq
.....................................................gcaccauuugaaaucaguguu.......   1      0    seq
```
